# Dynamics of genome evolution in facultative symbionts of aphids

**DOI:** 10.1111/j.1462-2920.2009.02085.x

**Published:** 2010-08

**Authors:** Patrick H Degnan, Teresa E Leonardo, Bodil N Cass, Bonnie Hurwitz, David Stern, Richard A Gibbs, Stephen Richards, Nancy A Moran

**Affiliations:** 1 Department of Ecology and Evolutionary Biology, University of ArizonaTucson, AZ 85721, USA; 2 Howard Hughes Medical Institute and Department of Ecology and Evolutionary Biology, Princeton UniversityPrinceton, NJ 08544, USA; 3 Center for Insect Science, University of ArizonaTucson, AZ 85721, USA; 4 Human Genome Sequencing Center, Baylor College of MedicineHouston, TX 77030, USA

## Abstract

Aphids are sap-feeding insects that host a range of bacterial endosymbionts including the obligate, nutritional mutualist *Buchnera* plus several bacteria that are not required for host survival. Among the latter, ‘*Candidatus* Regiella insecticola’ and ‘*Candidatus* Hamiltonella defensa’ are found in pea aphids and other hosts and have been shown to protect aphids from natural enemies. We have sequenced almost the entire genome of *R. insecticola* (2.07 Mbp) and compared it with the recently published genome of *H. defensa* (2.11 Mbp). Despite being sister species the two genomes are highly rearranged and the genomes only have ∼55% of genes in common. The functions encoded by the shared genes imply that the bacteria have similar metabolic capabilities, including only two essential amino acid biosynthetic pathways and active uptake mechanisms for the remaining eight, and similar capacities for host cell toxicity and invasion (type 3 secretion systems and RTX toxins). These observations, combined with high sequence divergence of orthologues, strongly suggest an ancient divergence after establishment of a symbiotic lifestyle. The divergence in gene sets and in genome architecture implies a history of rampant recombination and gene inactivation and the ongoing integration of mobile DNA (insertion sequence elements, prophage and plasmids).

## Introduction

Insect symbionts are widespread and diverse microbes that can provide essential nutrients to their hosts, manipulate their sex ratios or protect them from natural enemies (Buchner, 1965; Moran *et al.*, 2008). The pea aphid, *Acyrthosiphon pisum*, and its bacterial symbionts are central models for the study of the evolution, origin and function of heritable symbioses. *Buchnera aphidicola* (*Gammaproteobacteria*) is an anciently acquired, vertically transmitted, coevolved obligate mutualist present in almost all aphids, which provides aphids with essential amino acids (Munson *et al.*, 1991; Shigenobu *et al.*, 2000). Aphids, including the pea aphid, can also contain a variety of other heritable bacterial symbionts that are not required for host growth and reproduction and that sometimes are transmitted horizontally (Sandström *et al.*, 2001; Russell *et al.*, 2003). These are referred to as ‘facultative’ or ‘secondary’ symbionts.

Among these facultative symbionts, ‘*Candidatus* Regiella insecticola’ and ‘*Candidatus* Hamiltonella defensa’ are common in aphids and have been shown to provide protection to aphids from natural enemies. *Hamiltonella defensa* confers resistance to parasitoid wasps (Bensadia *et al.*, 2005; Oliver *et al.*, 2003; 2005). *Regiella insecticola* provides protection from fungal pathogens (Scarborough *et al.*, 2005) and may also protect against parasitoid wasps (von Burg *et al.*, 2008). In pea aphids, *R. insecticola* infection is correlated with use of clover as a host plant (Tsuchida *et al.*, 2002; Leonardo and Miuru, 2003; Simon *et al.*, 2003; Ferrari *et al.*, 2004), and one experimental study indicated that *R. insecticola* improves host performance on that host plant (Tsuchida *et al.*, 2004; but see Leonardo, 2004). Finally, *R. insecticola* is associated with changes in the timing of sexual induction response and with alteration of the wing induction response (Leonardo and Mondor, 2006), likely as the by-product of other effects on host physiology. *Hamiltonella defensa* strains vary in their effects on aphid hosts (Oliver *et al.*, 2005), and such strain variation is likely present in *R. insecticola.*

Phylogenies based on sequences of conserved genes support the status of *H. defensa* and *R. insecticola* as sister species within the *Enterobacteriaceae* (*Gammaproteobacteria*) (Degnan and Moran, 2008a). These two species show similar lifestyles. Both infect many aphid species where they reside in the hemolymph, in cells surrounding the primary bacteriocytes that contain *B. aphidicola*, and in bacteriocytes themselves (Moran *et al.*, 2005; Tsuchida *et al.*, 2005). Although routinely vertically transmitted through the ovaries of female aphids, both symbionts are abundant in male accessory glands and can be sexually transmitted (Moran and Dunbar, 2006). Their host range beyond aphids has not been thoroughly assessed, although *H. defensa* has been detected in some other insect species (Russell *et al.*, 2003). The most intensive surveys have been in pea aphid populations, where both species exhibit variable infection frequencies (16–70%) (Sandström *et al.*, 2001; Tsuchida *et al.*, 2002; Leonardo and Miuru, 2003; Simon *et al.*, 2003; Ferrari *et al.*, 2004).

Genomic approaches have offered a useful window into the evolution, ecology and function of symbionts. The pea aphid provides a first system in which genomes of a host (International Aphid Genomics Consortium. 2009), its obligate symbiont *B. aphidicola* (Shigenobu *et al.*, 2000; Moran *et al.*, 2009) and one facultative symbiont, *H. defensa* (Degnan *et al.*, 2009), have been completed. Here we report essentially the entire *R. insecticola* genome, obtaining significant insight into the lifestyle and metabolic interaction of this second facultative symbiont with aphids and *B. aphidicola*. By comparing the *R. insecticola* and *H. defensa* genomes, we can elucidate the evolutionary patterns characterizing facultative symbiont genomes, which serve as portals for novel heritable traits within their hosts.

## Results

We have assembled and annotated 2.07 Mbp of unique sequence from *R. insecticola* str. LSR1 (Table 1). The sequence consists of five large scaffolds, totalling 1.48 Mbp, reconstructed from sequenced BACs and 454 pyrosequencing data (Fig. S1) plus 168 shorter contigs, totalling 0.59 Mbp, assembled solely from 454 data. PCR amplicons and Sanger sequencing were used to close four gaps. Several lines of evidence indicate that essentially all unique sequence in the *R. insecticola* genome is represented. First, genome-wide 454 coverage of single-copy regions averages 11-fold, a level expected to result in representation of essentially all genes (Table S1). Second, pulsed-field gel electrophoresis of digested *R. insecticola* chromosomes supports a genome size of approximately 2.1–2.4 Mbp (data not shown). Because the genome includes some multicopy sequences (of mobile elements) that are partially collapsed during assembly, the actual genome size is expected to be slightly larger than our 2.07 Mbp of unique sequence. Third, our sequence includes 204 of 205 genes known to be present as single-copy genes in almost all *Gammaproteobacteria* (Lerat *et al.*, 2003). Thus, the lack of complete assembly primarily reflects the abundance of repetitive sequence rather than incomplete sequencing. This exhaustive coverage allows robust interpretations of *R. insecticola*'s metabolic and other functional capabilities.

**Table 1:** Comparison of some general features of the *Regiella insecticola* genome with other bacteria.

	*B. aphidicola* APS	*H. defensa* 5AT	*R. insecticola* LSR1	*S. glossindius*	*E. coli* K12	*Y. pestis* CO92
Chromosome, bp	640 681	2 110 331	2 035 106	4 171 146	4 639 221	4 653 728
Extrachromosomal elements	2	1 (59 032)	1 (32 491)	3	–	3
Total G + C (%)	26.2	40.1	42.4	54.7	50.8	47.6
Total predicted CDS	571	2 100 (56)	1 761 (27)	2 432	4 284	4 012
Coding density (%)	86.7	80.8	71.4	50.9	87.9	83.8
Average CDS size (bp)	984	812	856	873	950	998
Pseudogenes	13	188 (1)	214 (3)	972	150	149
rRNA operons	2	3	4	7	7	6
tRNAs	32	42	36	69	86	70
Lifestyle	Obligate	Facultative	Facultative	Facultative	Commensal	Pathogen

We identified 1788 coding sequences (CDS) plus 217 pseudogenes in the scaffolds. Of the CDS, 88.1% have a significant database match (Fig. S2). The mean CDS length and coding density of the scaffolds are lower than observed in most bacteria due to mobile DNA activity resulting in gene truncations and inactivations (pseudogenes) (Table 1). Consistent with previous estimates (Degnan and Moran, 2008a), the *R. insecticola* scaffolds are moderately A + T biased (42.4% GC).

### Genome comparison: *R. insecticola – H. defensa*

The shared genome content of *R. insecticola* and *H. defensa* was determined utilizing a reciprocal best hit (RBH) strategy. Although both genomes contained a collection of genes for which one-to-one orthologues could not be identified, a total of 918 intact single-copy orthologues (SICO) were found (*Ri-Hd*) (Table 2). A second round of Blast searches was performed using the pseudogenes from each genome to detect potential lineage-specific and shared gene inactivations (Table 2). Considering only intact genes, the two species share approximately 55% of their genes. The majority of the genes unique to *R. insecticola* comprise conserved hypothetical CDS (*n* = 101), non-conserved hypothetical CDS (*n* = 234) and multicopy transposase CDS (*n* = 54).

**Table 2:** Summary of genes and pseudogenes shared by *R. insecticola* and *H. defensa* and unique to each.

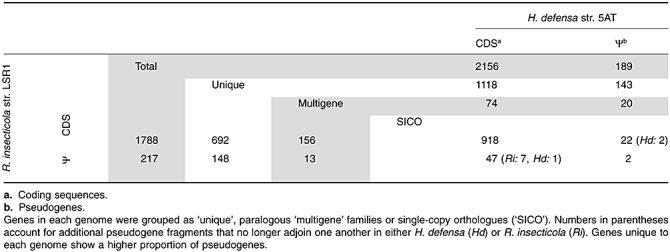

**Fig. 1:**
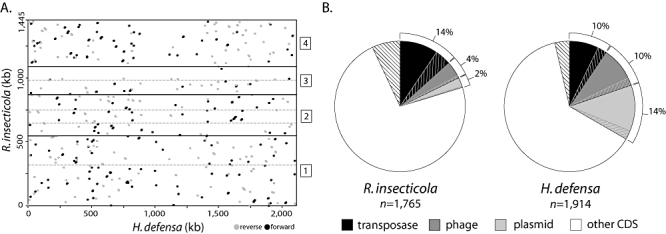
Comparing gene arrangements and mobile gene complement for *R. insecticola* and *H. defensa*. A. Co-ordinates and orientations of RBH between *R. insecticola* and *H. defensa* are plotted against each other, and show almost no colinearity between the two endosymbiont genomes. Solid black horizontal lines demark the ends of the four genome scaffolds, and horizontal grey dotted lines the physical and sequence gaps in each scaffold. B. Distributions of intact genes and pseudogenes in the three major classes of mobile DNA in *R. insecticola* and *H. defensa*. Gene numbers include plasmid encoded genes and pseudogenes, but exclude strictly hypothetical genes. Percentages indicate the fraction of each category, including pseudogenes (hatched).

The extent of genome colinearity (synteny) between these sister taxa is low and is limited to a few clusters of genes, based on comparisons of the genome co-ordinates and strand for orthologues (Fig. 1A). Nearly a third of the 711 orthologues present on *R. insecticola* BACs exist as singletons, and only 16 conserved gene blocks are six genes or longer (Fig. S3).

Estimates of non-synonymous substitutions per non-synonymous site (dN) and synonymous substitutions per synonymous site (dS) were calculated for each orthologue pair. The mean dN is 0.262 ± 0.138, while dS is saturated (>> 3.00). Of the 647 *Ri-Hd* SICO genes identified from the sequenced BAC clones, 199 are conserved in *Buchnera* spp., *Blochmannia* spp., *Escherichia coli* and *Salmonella typhimurium* LT2. Non-synonymous divergence at these loci was correlated with non-synonymous divergence values for these other genome pairs (*P*< 0.0001, Spearman's rho, Fig. S4) and is consistent with mostly vertical transmission of these genes down lineages.

Thus, genomes of *R. insecticola* and *H. defensa* retain a large set of shared orthologues but have undergone extensive sequence divergence and extensive rearrangements. Although these two symbionts are known to sometimes co-occur in the same hosts, most orthologous genes show no sign of recombination or exchange between these genomes (but see section on plasmid genes below).

### Metabolic reconstruction of *R. insecticola*

The *R. insecticola* and *H. defensa* show striking similarity in the central metabolism, transport and biosynthetic machinery inferred from their gene sets (Table S2)*.* Both *H. defensa* (Degnan *et al.*, 2009) and *R. insecticola* possess much of the aerobic respiratory chain, including glycolysis, the TCA cycle, cytochrome *bo* oxidase, ATP synthase and NADH dehydrogenase I and anaerobic fermentation of phosphoenolpyruvate and acetyl-CoA. Although both symbionts have retained the glucose-specific phosphotransferase system, each has a unique set of additional mechanisms for the acquisition of carbon compounds (Table S2). *R. insecticola* encodes NADH dehydrogenase II and an inactivated cytochrome *bd* oxidase that are not found in *H. defensa*.

*Regiella insecticola* resembles *H. defensa* in having lost all essential amino acid biosynthetic pathways except those for threonine and lysine, while retaining the complementary active transporters (Degnan *et al.,* 2009). Several genes involved in amino acid synthesis or acquisition that are absent in *H. defensa* and present in *R. insecticola* are those for the uptake of glutamate (*gltS*), alanine, serine and glycine (*cycA*), cysteine (*cydCD*) and the biosynthesis of glutamine from glutamate and ammonia (*glnA*). This latter pathway potentially affects the nitrogen budget of the host by providing a route for recycling waste nitrogen. *Regiella insecticola* and *H. defensa* also share a similar suite of genes involved in vitamin and cofactor biosynthesis (coenzyme A, isoprenoids, ubiquinone, B_2_, B_3_, B_6_, B_9_) and transport (pantothenate). The presence of pseudogenes for *bioCDH*, *speD, thiP* and *hmuVR* in *R. insecticola* suggest loss of the ability to synthesize biotin or spermidine and to take up thiamine (B_1_) and hemin. The inability of *R. insecticola* to import hemin or thiamine however, seems unimportant, as the entire biosynthetic pathways for both metabolites are intact. Additional differences in regulation, cellular processes, transport of inorganic ions and DNA repair, replication and recombination between *R. insecticola* and *H. defensa* are expected given the pseudogenes and unique genes in *R. insecticola* (Table S2).

### Genomic islands in *R. insecticola*

Among the genes present in *R. insecticola* and absent from *H. defensa* are 18 genes encoding components and regulators of the flagellar apparatus, present as several genomic clusters (Table S3). Most of the essential genes for producing a flagellum are present. These include *lafA*, encoding lateral flagellin, but not *fliC*, the typical flagellin. Based on studies in some other *Gammaproteobacteria*, LafA is implicated in swarming on solid or viscous media rather than movement through liquid media (McCarter, 2004). Flagella have not been observed in the few available electron micrographs of *R. insecticola* (Moran *et al.*, 2005; Tsuchida *et al.*, 2005). Potentially, *R. insecticola* uses a flagellum at a particular life cycle stage, such as transmission.

**Fig. 2:**
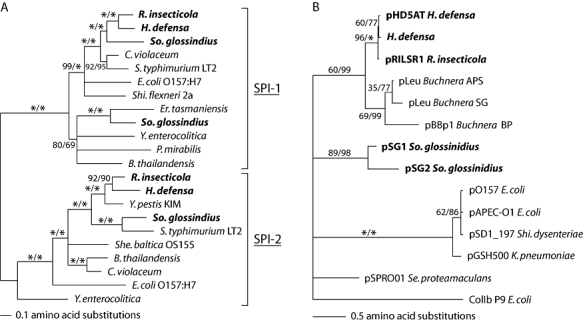
Phylogenies of T3SS and plasmid genes in insect endosymbionts. A. *R. insecticola* is among three facultative endosymbionts (shown in bold) known to encode a T3SS. Both SPI-1- and SPI-2-like T3SS of *R. insecticola* are sister to those in *H. defensa*. B. Phylogeny of IncFII family plasmid replication proteins (RepA) from plasmids of *Regiella, Hamiltonella, Buchnera* and *Sodalis*. Best trees generated by RAxML and bootstrap scores estimated from 100 non-parametric bootstrap replicates in RAxML and PhyML. Values of 100 are shown as asterisks (*) and branches with < 70 bootstrap support by both methods are collapsed. Genus abbreviations are as follows: *S., Salmonella, C., Chromobacterium, So., Sodalis, E., Escherichia, Shi., Shigella, B., Burkholderia, Y., Yersinia, P., Proteus, Er., Erwinia, R., Regiella, She, Shewanella, K., Klebsiella, Se., Serratia*.

*Regiella insecticola* also carries two type 3 secretion systems (T3SS) similar to SPI-1 and SPI-2 from *S. typhimurium* LT2 and *H. defensa.* Indeed phylogenetic reconstruction using a concatenation of six conserved T3SS structural proteins suggests that the ancestor of *R. insecticola* and *H. defensa* also encoded these T3SS (Fig. 2A), an inference that is supported by the observation that the sequence divergence for these genes is near the genome average. The SPI-1 locus no longer appears functional as it has been broken into a minimum of four fragments and fewer than half of the essential genes were identified. Although the SPI-2 locus has also been fragmented into two blocks, it has all of the core genes and is likely to still be active. Both T3SS in *H. defensa* have also been split into two, which is unusual. However, these recombination events appear to have occurred independently in these symbiont lineages. Six putative T3SS effector proteins including two adenylate cyclases were also found on the *R. insecticola* scaffolds. Other putative virulence factors were identified, including five CDS with similarity to MCF toxins, one YD-repeat containing toxin and 11 RTX toxins. This last toxin family is also abundant in *H. defensa*, and it would appear that it has also undergone divergence and inactivation in *R. insecticola*.

### Mobile DNA

Similar to *H. defensa* (Degnan *et al.*, 2009), *R. insecticola* possesses an abundance of mobile DNA (transposase, prophage and plasmids) (Fig. 1B). Insertion sequence (IS) elements are the dominant type of mobile DNA, representing 14% of non-hypothetical CDS and pseudogenes; more than a third of these belong to a single repeat type (Table S4). Three *R. insecticola* IS elements and one *H. defensa* IS element are similar to single copy inactivated or partial elements found in the other genome. These pairs have > 75% nucleotide identity and in each case at least one of the inverted repeats is conserved. The IS elements in conjunction with DNA recombination mechanisms probably have contributed to the extensive genome reorganization in *R. insecticola* and *H. defensa* (Fig. 1A).

No intact prophage infects the sequenced strain of *R. insecticola*, in contrast to the case for *H. defensa* (Degnan *et al.*, 2009). Nine partial prophage islands (0.6–14 kb) were identified in the scaffolds (Table S3). These regions have somewhat elevated G + C base composition (44%) and were frequently found adjacent to tRNA genes or flanked by IS elements. The IS elements also appear to have played a role in prophage inactivation and recombination.

Plasmid-associated genes were relatively few among the *R. insecticola* scaffolds except for scaffold number 5 and two 454 contigs (Fig. S1, Table S3). The BACs representing scaffold 5 were significantly shorter than the other finished BACs, no paired BES linked it to any other scaffold and PCR verified that it forms a circular 32.5 kb plasmid which we designated pRILSR1. The pyrosequencing coverage was fourfold higher than the genome average (Table S1), consistent with its presence as a low-copy-number plasmid. The genes encoded on pRILSR1 comprise a complete *virB*-like type IV conjugative system similar to pFBAOT6 from *Aeromonas punctata*. Interestingly, the pRILSR1-encoded *traC* gene, which is essential for the sex pilus formation, is disrupted through frameshift deletion. The annotated genes on the 454 contigs are similar to the type IV conjugative system of *Erwinia amylovora* pEU30.

*Hamiltonella defensa* str. 5AT also carries an intact type IV conjugative plasmid (pHD5AT), as well as four integrated and inactivated type IV conjugative plasmids (Degnan *et al.*, 2009). The pRILSR1 type IV conjugative system is only distantly related to those in *H. defensa*, but the *repA* genes of pRILSR1 and pHD5AT are very similar (dN = 0.0217, dS = 0.2597) (Fig. 2B) as compared with the average divergence for orthologues. The other type IV conjugative system genes in *R. insecticola* were identified as SICO of genes in *H. defensa* plasmid island 2. Estimates of dN and dS for these genes are also lower than the genome average (mean dN = 0.095, mean dS = 0.952). This strongly implies a more recent divergence due to horizontal transfer of these plasmid genes between *R. insecticola* and *H. defensa* after the species divergence.

## Discussion

### Trends in aphid facultative endosymbiont genomes

*Regiella insecticola* and *H. defensa* are both heritable facultative symbionts of aphids, occasionally horizontally transmitted and protective against natural enemies of aphid hosts. Moreover, they are sister species, based on phylogenetic analyses of several loci (Sandström *et al.*, 2001; Russell *et al.*, 2003; Darby *et al.*, 2005; Moran *et al.*, 2005; Degnan and Moran, 2008a; Degnan *et al.*, 2009). Thus, common ancestry, rather than convergence, is the likely basis for the similarities in both lifestyle and genome content. Because the limited biosynthetic abilities imply restriction to a host, we hypothesize that this ancestor was a host-restricted aerobic heterotroph taking advantage of the abundant sugars in the host diet of plant sap and the essential amino acids produced by the obligate symbiont.

Similarities of *R. insecticola* and *H. defensa* extend to pathogenicity factors. In particular, both genomes encode T3SS, which are presumably used to gain access to aphid cells. Experimental evidence supports this role for the T3SS in the symbionts of tsetse flies and weevils (Dale *et al.*, 2001; 2002). Although T3SS genes are known to be horizontally transferred in *Bacteria*, as reflected in the lack of correspondence between the T3SS gene trees and bacterial species phylogeny (Fig. 2A), several observations indicate that the copies in *R. insecticola* and *H. defensa* appear to be continuously present from the time of their shared ancestor. First, these genes display G + C content similar to that of their respective genomes, and estimates of dN (mean dN = 0.324) are comparable to estimates for single-copy orthologous genes (mean dN = 0.262). Furthermore, phylogenetic analyses of genes in the SPI-1- and SPI-2-like T3SS support common ancestry of these systems in *R. insecticola* and *H. defensa* (Fig. 2A). Divergence among putative T3SS effectors and some other putative toxins is high, complicating phylogenetic reconstruction, but both genomes contain homologues of virulence factors that target eukaryotic cell functions. Expression and utilization of these toxins has not been demonstrated, but their maintenance in light of highly disrupted genomes (pseudogenes, IS activity) argues for their utility and functionality.

Together, these observations strongly support the view that the common ancestor of *R. insecticola* and *H. defensa* was an insect symbiont that used T3SS and other effectors as a mechanism for host invasion. The high sequence divergence of orthologues implies that this shared ancestor was quite ancient. For example, silent divergence (dS) values are saturated and thus higher than those for *E. coli*–*S. typhimurium*, a divergence that is estimated at 100 My (Ochman and Wilson, 1987). Because the rate of sequence evolution is elevated in these symbionts (Russell *et al.*, 2003), and there is no calibration point for a molecular clock, reliable estimates of a date for the *R. insecticola*–*H. defensa* ancestor are not possible. Rates are also elevated in *B. aphidicola* and the *R. insecticola*–*H. defensa* divergence level is of the same order as that between *B. aphidicola* that diverged with aphid hosts 60–200 My. Thus, even conservative calculations would put the *R. insecticola*–*H. defensa* divergence at many millions of years, implying that the comparison of these two genomes gives a picture of the evolutionary changes that accompany the long-term evolution of facultative symbiont lineages.

### Large and dynamic pools of mobile DNA

Both *R. insecticola* and *H. defensa* genomes are overrun with mobile DNA (Fig. 1B), a feature that contrasts with the genomes of obligate symbionts such as *B. aphidicola* (Tamas *et al.*, 2002; van Ham *et al.*, 2003). Although both genomes feature large numbers of transposases and phage- or plasmid-associated genes, the actual gene sets involved are largely genome-specific. This distinctiveness has persisted in spite of *R. insecticola* and *H. defensa* frequently coinfecting the same individual aphids (Sandström *et al.*, 2001; Simon *et al.*, 2003; Ferrari *et al.*, 2004) where they reside in bacteriocytes, hemolymph and embryos (Tsuchida *et al.*, 2005; Moran *et al.,* 2005), a situation that would appear to create the potential for horizontal gene exchange.

The genome content of *R. insecticola* and *H. defensa* can be divided into two distinct categories of genes with respect to involvement in recombination and transfer. The ‘core’ set underlying basic cellular processes and metabolism (Table S2) is highly stable and appears to be inherited clonally, based on analyses of *H. defensa* strains (Degnan and Moran, 2008a) and on the uniform distance of orthologues between *H. defensa* and *R. insecticola*. The mobile portion of the genome is dynamic and has diverged extensively between the genomes.

Only a small fraction of *R. insecticola* genes (128/2005) exhibit evidence of recent horizontal exchange with *H. defensa*, using the criterion of dS < 1.0. Most of the recently transferred genes consist of plasmid-related genes (*n* = 9) and copies of the ISRin1 multicopy IS element (*n* = 110). There is no evidence for recent exchange of phage-related genes. The clear lack of homologous recombination between *R. insecticola* and *H. defensa* is consistent with other evidence that these organisms comprise two distinct symbiont species that have diverged sufficiently that homologous recombination is precluded. The lack of extensive HGT likely reflects a combination of factors including host ranges of conjugative plasmids and phage and innate restriction modification and exclusion mechanisms.

Similar to *H. defensa* and *R. insecticola*, the reproductive parasites of arthropods, *Wolbachia pipientis* str. *w*Mel, *w*Pip and *w*Ri, are also facultative symbionts of insects. Comparisons among the genomes of these *Wolbachia* strains reveal similarities to the comparison of *H. defensa* and *R. insecticola* genomes. For example, both symbiont lineages exhibit extensive amounts of mobile DNA, yet maintain a core set of metabolic pathways (Klasson *et al.*, 2008). In contrast to the clonal evolution of core genes in *H. defensa* and *R. insecticola*, the *Wolbachia* genomes display a wide range of dS values across genes, indicating ongoing gene exchange and recombination of homologous genes, probably in the context of coinfections (Klasson *et al.*, 2009). Furthermore, transfer and recombination of phage are rampant between *Wolbachia* supergroups (Bordenstein and Wernegreen, 2004) and has resulted in sequenced *Wolbachia* genomes containing two to five integrated copies of phage WO (Klasson *et al.*, 2008).

The uniform and high sequence divergence of core genes for the *H. defensa* and *R. insecticola* comparison resembles patterns of divergence observed in pairs of obligate symbionts such as *B. aphidicola* from different aphid species (Tamas *et al.*, 2002; van Ham *et al.*, 2003) and *Blochmannia* from different ant species (Degnan *et al.*, 2005). However, *H. defensa* and *R. insecticola* possess distinct sets of mobile elements and have diverged in gene order. Whereas the obligate symbionts, *B. aphidicola*, show the most stable genome architecture of any bacteria, with no rearrangements over periods up to 150 My, *H. defensa* and *R. insecticola* are at the opposite extreme. *Wolbachia pipientis* strains *w*Mel and *w*Pip, also have undergone massive genome reorganization but show low estimates of nucleotide divergence (Baldo *et al.*, 2006; Klasson *et al.*, 2008). Thus, *R. insecticola* and *H. defensa* have estimates of nucleotide divergence similar to obligate mutualists, but with levels of recombination and rearrangement similar to the obligate parasites such as *Wolbachia*.

### Outlook on *R. insecticola* genomics

*Regiella insecticola* is common among pea aphids, and it infects other aphid genera in the tribe Macrosiphini as well as aphids from other tribes (Sandström *et al.*, 2001; Russell *et al.*, 2003; Haynes *et al.* 2003). Given its wide distribution and large fraction of mobile DNA we expect high levels of genomic variation among strains. Such genetic variation, including phage, plasmids or other genomic islands, probably underlies variation in the described phenotypes (Tsuchida *et al.*, 2004; Scarborough *et al.*, 2005; von Burg *et al.*, 2008). This is a pattern shared with *H. defensa*, in which gene content differences are implicated in differences in protection from parasitoids (Degnan and Moran, 2008b; Oliver *et al.*, 2009). Similarly, a link between mobile genetic element content and capacity for reproductive manipulation has been suggested for *W. pipientis* (Sinkins *et al.* 2005). Availability of genome sequences for *R. insecticola* and other aphid symbionts (Shigenobu *et al.*, 2000; Degnan *et al.*, 2009), and for the pea aphid host (International Aphid Genomics Consortium, 2009) will facilitate further efforts to interrogate host–symbiont interactions.

## Experimental procedures

### Sequencing of LSR1 BAC library

Preliminary analysis of the *A. pisum* str. LSR1 BAC library (APP_Ba: Clemson University Genomics Institute) indicated that a significant fraction contained inserts from both the obligate aphid endosymbiont *B. aphidicola* and the facultative endosymbiont *R. insecticola*. Using PCR specific for *R. insecticola* and fingerprint analysis of BAC inserts, a tiling path of potential *R. insecticola* BACs was generated. Selected BACs were subcloned and Sanger sequenced on 3730 sequencers (Applied Biosystems, Foster City, CA) and assembled using the Atlas assembly pipeline at the HGSC. Initial overlaps and redundancy in the BAC contigs were assessed using BlastN. Subsequently, BAC contigs were assembled into supercontigs with Phrap (phrap.org). Scaffolds of the supercontigs were formed using the order and orientation of paired-end sequence reads from 1140 APP_Ba BACs (Fig. S1).

Coverage of the *R. insecticola* genome was extended using 454 FLX pyrosequencing. A sample of purified *R. insecticola* cells from *A. pisum* strain LSR1 with the original *R. insecticola* infection was obtained and DNA purified using methods previously applied to *H. defensa* (Degnan *et al.*, 2009). 454 library construction and sequencing were carried out at the HGSC. Pyrosequencing reads were assembled with Newbler (v 2.0.0), and contigs were sorted and identified using Blast,% G + C and read depth as *R. insecticola*, *B. aphidicola*, mitochondrial or from the pea aphid (Table S1). Several BAC supercontigs were merged and extended using the Newbler contigs and Sanger sequencing of PCR products (Fig. S1) (as in Degnan *et al.*, 2009).

### Gene prediction and annotation

All contigs were analysed with both Glimmer v2.13 and v3.02 to identify predicted open reading frames (ORFs) (Delcher *et al.*, 1999). These algorthims were implemented using either a training set of 93 *R. insecticola* genes previously identified (P.H. Degnan, unpublished) or the g3-iterate.csh script respectively. The predicted ORFs from each search were then reduced to a non-redundant set. To further minimize the chance of missed genes or inactivated pseudogenes, intergenic spacers were screened using BlastX against the NR database. The final set of putative ORFs was then annotated using evidence from similarity searches using BlastP (NR, COGs, *E. coli*) and Hmmr (Pfam_ls, TIGRFAM8.0) (Bateman *et al.*, 2004). Search results were filtered requiring > 50% coverage and expectation values < 1e^−10^ for BlastP, and expectation values < 0.1 for Hmmr. Gene products were assigned to ORFs only when the search results were unambiguous, and ORFs with conflicting or ambiguous similarity results were annotated as putative. The ORFs that were > 30 amino acids and did not significantly overlap adjacent ORFs but lacked a significant database match were annotated as hypothetical. Final manual inspection identified both adjacent ORFs representing fragments of the same gene and truncated ORFs (< 60% length of homologues) and annotated these cases as pseudogenes. Non-coding RNA genes were identified using BlastN (rRNAs) and tRNAscan-Se (tRNAs) (Lowe and Eddy, 1997). Multicopy, mobile genetic elements were initially identified based upon recurring, identical ORFs. Sequences flanking each putative transposase type were extracted and aligned using Mafft (Katoh and Toh, 2008), and boundaries and/or inverted repeats of each mobile genetic element were determined.

### Comparative genome analyses

Previous research had identified another facultative endosymbiont of aphids, *H. defensa*, to be the most closely related bacterial lineage to *R. insecticola* (Sandström *et al.*, 2001). Thus we first identified the shared, intact orthologues between *R. insecticola* and *H. defensa* (*Ri-Hd*) using BlastP to identify RBH. We used an expectation value cut-off of 1e^−30^ and an 80% aligned length cut-off. The RBH pairs were manually inspected and several multigene families were identified and spurious matches were discarded. The identities and functions of the remaining non-orthologous genes from each genome were then assessed (Karp *et al.*, 2007), as was the potential presence of inactivated copies (pseudogenes). The locations and orientations of the RBH in the two genomes were then compared to analyse the extent of genome colinearity. Because this analysis required high-quality evidence regarding gene arrangements, we included only the *R. insecticola* genes on the larger scaffolds, which were based on the BAC sequencing; this comprised approximately 75% of the genome.

The *Regiella* CDS were also compared with seven other genomes of free-living and obligately endosymbiotic bacteria; *E. coli* K12, *S. typhimurium* LT2, *Blochmannia floridanus*, *Bl. pennsylvanicus*, *B. aphidicola* Aps, *Bu. aphidicola* Bp and *Bu. aphidicola* Sg. The *Ri-Hd* set of orthologues was searched against the seven genomes using BlastP and orthologues were identified using a bit score cut-off ratio of ≥ 0.3 (as in Lerat *et al.*, 2003). Pairwise alignments of the genes common to all nine genomes were generated among various genome pairs. Nucleotide sequences were aligned based on Mafft alignments of their protein translations. All gaps and stop codons were removed from the alignments, and pairwise estimates of non-synonymous substitutions per non-synonymous site (dN) and synonymous substitutions per synonymous site (dS) were calculated using the method of Goldman and Yang (1994) in Paml (Yang, 1997). All statistical comparisons of pairwise estimates of dN were performed in JMP (SAS Institute).

### Phylogenetic reconstruction

Sequences for homologues of the *R. insecticola* and *H. defensa* T3SS genes (*ssaN/invC, ssaR/spaP, ssaS/spaQ, ssaT/sparR, ssaU/spaS* and *ssaV/invA*) and incFII plasmid replication gene (*repA*) were retrieved from GenBank. The amino acid sequences were aligned with Mafft and ambiguous regions were removed. The six T3SS genes were concatenated into a single alignment. Phylogenies and non-parametric bootstraps were estimated using RAxML (Stamatakis, 2006) and PhyML (Guindon and Gascuel, 2003) as in Degnan and colleagues (2009).

## Supporting information

**Fig. S1.** Schematic of *R. insecticola* genome scaffolds. Five *R. insecticola* scaffolds were reconstructed from 24 partially and completely sequenced BACs. Consensus contig sequences are shown as black bars with lengths in kilobases. The BACs comprising each contig are shown below as grey bars and sequence gaps in the BAC sequences are shown as dotted grey lines. Sequence gaps closed by PCR are shown as solid black lines and extensions generated by pyrosequencing contigs are orange. Curved lines above the contigs indicate the number of pairs of BAC end sequences that are contained within (grey) or connect contigs (black).

**Fig. S2.** Schematic of annotated *R. insecticola* genome scaffolds from BAC sequences. The five annotated genome scaffolds represent 1.48 Mb of the *R. insecticola* genome. CDS on the forward strand are shown in grey above, reverse strand below also in grey. Several specific gene features are highlighted; ribosomal RNAs (red), pseudogenes (dark grey) and transposases (green) as well as phage (blue), plasmid (purple), flagellar (orange) and T3SS and virulence genes (pink). Vertical red lines show the locations of gaps in the scaffolds. The asterisk (*) denotes the origin of replication.

**Fig. S3.** Numbers of syntenic gene blocks for SICO genes of *R. insecticola* and *H. defensa*. Conservation of gene order (syteny) between BAC encoded *R. insecticola* SICO genes and their *H. defensa* orthologues was determined (*n* = 711). Genes were found as singletons (1) or in blocks of 2–28 genes. Numbers above bars indicate the number of blocks of a given length (black) and the number of genes in these blocks (grey).

**Fig. S4.** Correlated rates of protein divergence in pairs of conserved bacterial orthologues. Estimates of nonsynonymous substitutions per non-synonymous site (dN) were measured between genome pairs for 199 genes shared by nine bacteria; *R. insecticola (Ri), H. defensa (Hd), E. coli (Ec), S. typhimurium (St), Blochmannia floridanus (Bf), Bl. pennsylvanicus (Bp), Buchnera aphidicola APS (BapA), Bu. aphidicola SG (BapS) and Bu. aphidicola BP (BapB)*. These values for *Ri-Hd* orthologues when plotted against the orthologues from the four other genome pairs show a significant correlation in pairwise dN. Significance values were determined for Spearman's rho (ρ), a non-parametric test of association. *Ri-Hd* and Bf-Bp have the lowest divergence, while dN is slightly higher in *BapA-BapB*, lower in *BapA-BapS* and much lower in *Ec-St*.

**Table S1.** Identity of pyrosequencing contigs from *R. insecticola* str. LSR1.

**Table S2.** Comparison of *R. insecticola* and *H. defensa* genes sets underlying different metabolic capabilities.

**Table S3.** Genomic islands inserted into the *R. insecticola* str. LSR1 genome.

**Table S4.** Major classes of *R. insecticola* insertion sequences (IS) identified in BAC scaffolds.
